# The Laryngoscope

**Published:** 1860-07

**Authors:** 


The Laryngoscope.—M. Czermak, Professor of Physiology at Pesth, is at the present time in Paris, with the view of bringing into notice an instrument for exploring the interior of the larynx, and with which the vocal cords, the ventricles of the larynx, the rings and bifurcation of the trachea, and the Eustachian tubes, can be seen with the utmost facility. The laryngoscope may be said to be an English invention, for we read in Liston's "Practical Surgery," London edition, 1840, p. 417, under the head of ulcerated glottis: "A view of the parts may be sometimes obtained by means of a speculum—such a glass as is used by dentists—on a long stalk, previously dipped in hot water, introduced with its reflecting surface downward, and carried well into the fauces." It was not, however, until 1855, that this idea was acted upon, when Garcia, professor of music, made some valuable experiments to elucidate the physiology of the human voice, by causing the image of the larynx to be reflected from a mirror placed against the soft palate upon another mirror placed before him. Two years subsequently, Dr. Türck endeavored to apply the instrument to medical diagnosis, and to him and Dr. Czermak is due the merit of directing the attention of the profession to its use. Garcia and Dr. Türck employed the light of the sun in their experiments, but Prof. Czermak has made the instrument of greater utility by applying it by means of artificial light. The latter physician has, moreover, published a monograph on the subject.

The observer will at first be puzzled by the altered position of the parts in the reflected image, the component structures of the larynx being seen upside down; but the right side of the larynx is seen on the right of the instrument, and so of the left. The beginner had, therefore, better study an excised larynx, so as to accustom himself to the changed position of the parts. The mode of conducting the examination, writes the Berlin correspondent of the *Medical Times and Gazette*, May 5th, 1860, is as follows: "A metallic mirror, varying in size from six to fourteen lines in diameter, in shape either square with rounded edges, as recommened by Czermak, or oval, according to Türck's proposal, or, as it has been found very convenient by Dr. Levin, of Berlin, semi-circular, with a concave inferior margin, soldered to a slightly flexible metallic handle, is to be introduced into the well-opened mouth and fixed in such an angle against the uvula and soft palate as to throw incident luminous rays upon the larynx, and to reflect an image of the parts thus illuminated into the eye of the observer. To prevent the mirror becoming dim by condensation of vapor upon its surface, it is necessary to warm it previous to introduction by dipping it into hot water or holding the unpolished surface over the flame of a small spirit-lamp. Garcia made use of the direct rays of the sun in his experiments; as this source of illumination, however, is not always available, and, even if so, attended with obvious inconveniences in practice, Czermak proposes the use of a perforated concave mirror of 7-12″ focal distance, by which the light of an ordinary lamp can be concentrated upon the larynx-speculum, the eye of the observer being applied to the perforation. As the distinctness of the image will depend upon the brilliancy of the illumination employed, it will be found advantageous to concentrate the light of the lamp upon the concave mirror, by means of a powerful bi-convex lens. Dr. Levin, of this city, (Berlin,) has devised a highly convenient apparatus for this purpose, consisting of a tin tube carrying a convex lens of two and a half inches focal distance, and of about the same diameter, which, by means of a simple contrivance, can be fixed horizontally over an argand-lamp after the shade has been removed."

"The perforated concave reflector can either be held between the teeth of the observer, fixed on a suitable ivory handle, as recommended by Czermak, or attached to a large spectacle-frame, according to Stellwag's proposal, or it can be suspended from a support screwed to the corner of the table on which the lamp is placed. The latter contrivance will be found the most convenient for practical purposes."

"It will be most convenient to place the lamp to the right of the patient, who is to be examined in the sitting posture, his hands resting upon his knees, his body slightly advanced, and his head slightly reclining backward. According to Professor Traube's advice, the lamp, concave mirror, and larynx-speculum ought to be on the same level, and the angle formed by the rays incident upon and reflected from the concave mirror as acute as possible; on this account it will be wise to place the lamp a little behind the patient. The observer supports the head and chin of the patient with the left, and introduces the larynx-speculum with his right hand, looking through the perforation of the concave mirror, by means of which he illuminates the pharynx."

"By causing the patient to sound alternately the Roman vowels, a, e, the velum and uvula will be raised so as to admit of the mirror being introduced with greater facility. In pressing the speculum against the soft palate and uvula, great care must be taken to avoid touching the posterior wall of the pharynx, the palatine arches, and the base of the tongue, to prevent the supervention of vomiting and deglutition."

